# The germ cell marker *dead end* reveals alternatively spliced transcripts with dissimilar expression

**DOI:** 10.1038/s41598-019-39101-9

**Published:** 2019-02-20

**Authors:** Ana Carina Nogueira Vasconcelos, Danilo Pedro Streit, Anna Octavera, Misako Miwa, Naoki Kabeya, Goro Yoshizaki

**Affiliations:** 1 Tokyo University of Marine Science and Technology, Department of Marine Biosciences, Tokyo, 108-8477 Japan; 2 Federal University of Rio Grande do Sul, Department of Animal Science, Porto Alegre, 91540-000 Brazil; 3 The University of Tokyo, Department of Aquatic Bioscience, Tokyo, 113-8654 Japan; 4 Present Address: Johns Hopkins University, Institute for Nanobiotechnology, Whiting School of Engineering, Baltimore, Maryland 21218 USA

## Abstract

Since the late 19th century, the Amazon species *Colossoma macropomum* (tambaqui) has been exploited commercially and the climate change has contributed to decline in tambaqui numbers. Although germ cell cryopreservation and transplantation can help preserve the species’ genetic resources semipermanently, its germ cell behavior has not been analyzed to date. In this study, we isolated the tambaqui’s *dead end* gene (*dnd*) homolog (*tdnd*) and used it as a molecular marker for germ cells to obtain basic information essential for transplantation. The amino acid sequence showed 98% similarity and 53% identity with the zebrafish *dnd*. Phylogenetic analysis and the presence of consensus motifs known for *dnd* revealed that *tdnd* encodes the *dnd* ortholog and its transcript is detectable only in the testes and ovaries, showing a strong positive signal in oocytes and spermatogonia. The tambaqui possesses, at least, three different transcripts of *tdnd* which show dissimilar expression profile in undifferentiated and sexually mature animals, suggesting that they play distinct roles in germline development and they may influence the choice of donors for the cell transplantation study.

## Introduction

Eukaryotic gene expression control is complex and refined, and virtually all cells of multicellular organisms have the same expression potential. Selective gene expression occurs during cell differentiation, and cells with identical genetic information acquire different physiological characteristics and functions. Although most gene expression controls are performed at transcription and translation levels, their fine-tuning is performed by alternative processing of messenger ribonucleic acid (mRNA) precursors, generating different forms of mature mRNAs, depending on the cell type^1,2^.

Constitutive splicing is an RNA processing mechanism in which intragenic regions (introns) are removed and expressed regions (exons) are joined to form a mature RNA. Alternative splicing, a concept proposed for the first time in 1978^3^, is a deviation from this preferred sequence, where certain exons are skipped, resulting in various forms of mature mRNAs^4^. Alternative splicing explains the discrepancy between the number of genes encoding proteins and the number of different proteins produced. Alternative splicing of precursor mRNA is a crucial mechanism for expanding gene expression complexity and plays a substantial role in cellular differentiation and organism development. This important form of gene regulation contributes to gene expression control and increases protein diversity^2,5,6^. It is estimated that in the human genome, more than 95% of genes undergo alternative splicing^7–9^.

The *dnd* gene, first identified in the zebrafish^10^, is a germplasm component and encodes an RNA-binding protein crucial for the migration and survival of primordial germ cells (PGCs). In *dnd*-depleted zebrafish embryos, PGCs fail to acquire motility, lose specific marker gene expression, and die within 24 h postfertilization^10^. The Dnd protein has been shown to bind to the 3′-untranslated region of mRNAs to displace micro-RNAs (miRNAs) that bind to adjacent sites on the same mRNA^11^. The dnd protein is localized to perinuclear germ granules within PGCs, and several knock-down studies have confirmed the importance of *dnd* for adequate development of germ cells in teleost fish^10,12–18^. By *in situ* hybridization (ISH) with an antisense RNA probe, *dnd* expression in medaka^19^ and turbot^20^ adults was found in germ cells at premeiotic and meiotic stages of both sexes. In the Pacific bluefin tuna (BFT), localization of *dnd* mRNA is restricted to type A spermatogonia and is not detected in other differentiated spermatogenic cells^21^.

The use of *dnd* as a germ cell marker has been increasing in studies involving germ cell transplantation. This technology was developed in mammals in 1994^22,23^ and became a valuable approach to studying germ cell biology as well as conducting research in the fields of animal production, cell culture, reproductive medicine, and transgenic animal production^24–28^. The germ cell transplantation could also be used as an alternative to preserve genetic resources of important fish species, such as *Colossoma macropomum* from the Amazon and Orinoco river basins in northern South America. Popularly known as tambaqui, *C*. *macropomum* is the second-largest scaled fish in South America and is among the most cultivated Neotropical fish species in fish farms^29^. Currently, only 1% of the fish in the natural environment have the size allowed for fishing (i.e., 55 cm), showing that the species’ exploitation is above the maximum sustainable yield^30^.

To develop a specific molecular marker for further identification of germ cells, we isolated the tambaqui homolog of *dnd* (*tdnd*) and examined expression patterns in the gonads at different stages of development. In addition, we identified for the first time, three transcripts of *dnd* in the tambaqui and proposed that alternative splicing in this gene might play a role in germline development.

## Results

### Cloning of the *dnd* cDNA and phylogenetic analysis

**Figure 1:**
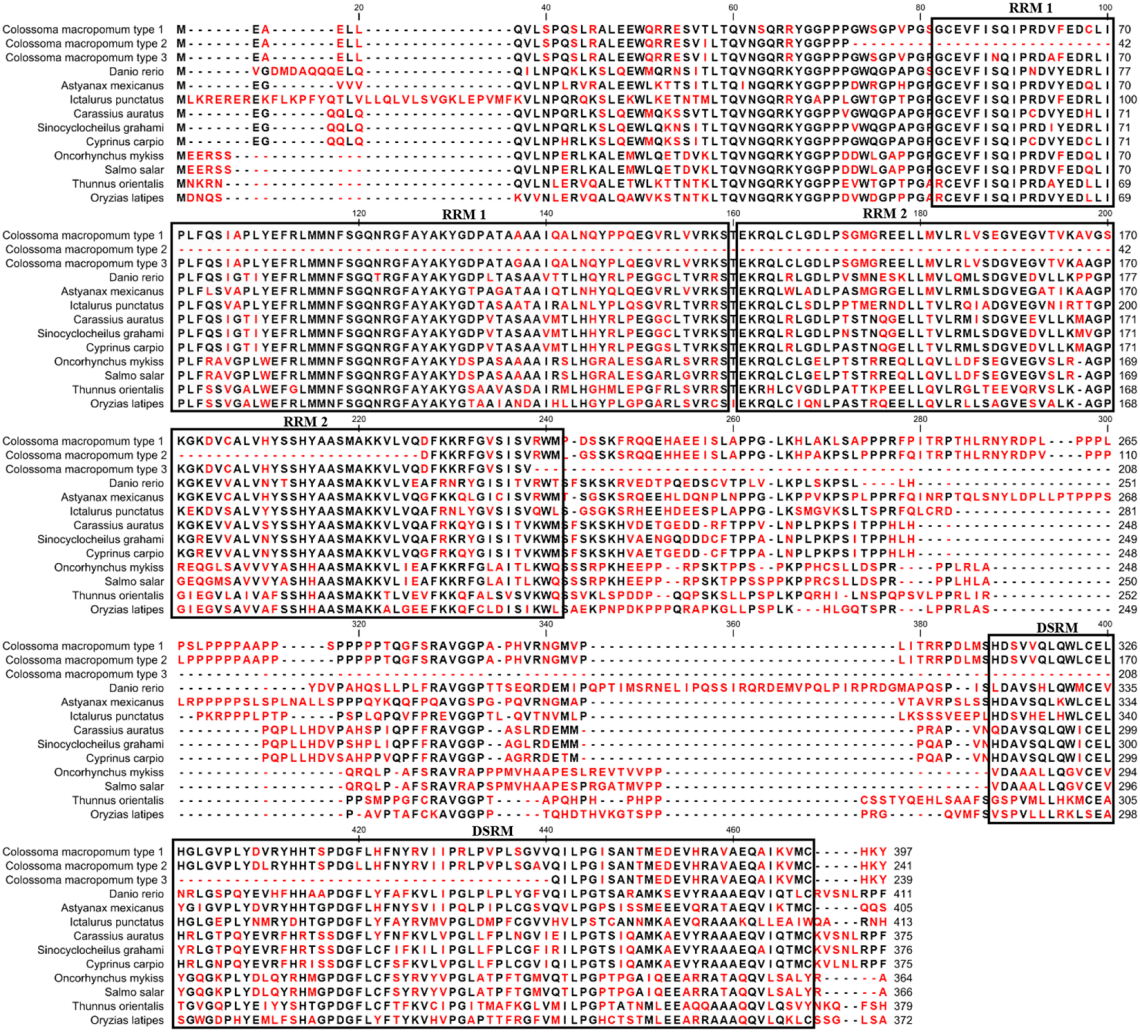
Multiple amino acid sequence alignment of tambaqui Dead end type1, 2 and 3, and other fishes Dnd proteins. The frames indicate the conserved motifs RRM (RNA recognition motif) and DSRM (double-stranded RNA recognition motif), while the red letter represents the non-conserved amino acids. The GeneBank IDs for Dnd homologues are as follow: *Colossoma macropomum* type 1 (AQY10189), type 2 (AST51843.1), and type 3 (AST51844.1); *Danio rerio* (NP_997960.1); *Astyanax mexicanus* (XP_007253662.1); *Ictalurus punctatus* (XP_017340221.1); *Carassius auratus* (AEX33122.1); *Sinocyclocheilus grahami* (XP_016098544.1); *Cyprinus carpio* (XP_018958879.1); *Oncorhynchus mykiss* (CDQ77433.1); *Salmo salar* (NP_001266060.1); *Thunnus orientalis* (KF128758.1); *Oryzias latipes* (NP_001157988.1).

**Figure 2:**
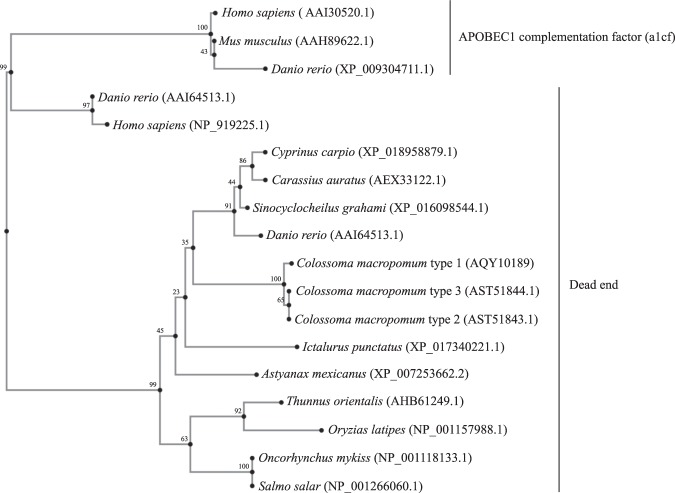
Maximum likelihood phylogenetic tree of Dead end proteins from different taxonomic groups. The GeneBank IDs for Dnd and APOBEC1 homologues are shown in front of each species. APOBEC1 complementation factor (a1cf) was included in the analysis and assigned as the out-group. The analyses were performed using the PhyML v3.0 server (Guindon *et al*.^52^). The protein evolutionary model was calculated in PhyML using smart model selection, resulting in LG + G + I, and the number of bootstrap replicates was set to 1000. The bootstrap values are presented as %.

The full length of *tdnd* (GenBank No. KY426013) has an open reading frame of 1194 bp, which begins with the start codon ATG at position 79 and ends at the stop codon TAA at position 1272. We inferred that the amino acid sequence encodes 398 amino acid residues and has three RNA recognition motifs (RRMs): RRM1 (AA 52–129), RRM2 (AA 131–211), and double-stranded RRM (DSRM) (AA 314–394) (Fig. 1). Among the three motifs, RRM1 is the most conserved (~82%), followed by the RRM2 (~76%) and the DSRM (~75%). The sequence alignment with other species indicated that *dnd* was highly conserved through the process of vertebrate evolution (Supplementary Table S1). Phylogenetic analysis comparing the amino acid sequences of the three isoforms of *tdnd* with those of related proteins from other species revealed that the *tdnd* sequence obtained in this study belongs to the clade of the *dnd* family (Fig. 2).

### Gene structure of *tdnd*

**Figure 3:**
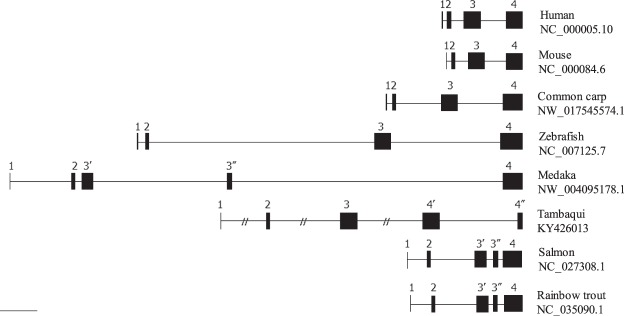
Schematic drawing of the exon–intron structure of various vertebrates with their respective GeneBank IDs. Predicted exons are shown in black boxes and introns as thin lines.

We analyzed the genomic DNA sequence of *tdnd* to verify the spliced site of the transcripts found in this study. After genomic DNA sequence analyzes and alignment with other species, we noticed that an exon of *tdnd* corresponds to exon 4 of all other known *dnd* homologs split into two exons and a new intron was inserted (Fig. 3, exons 4′ and 4″). Figure 3 shows the alignment of the *dnd* locus of various vertebrates. As shown, among these species, only the tambaqui has a segmentalized exon 4.

**Figure 4:**
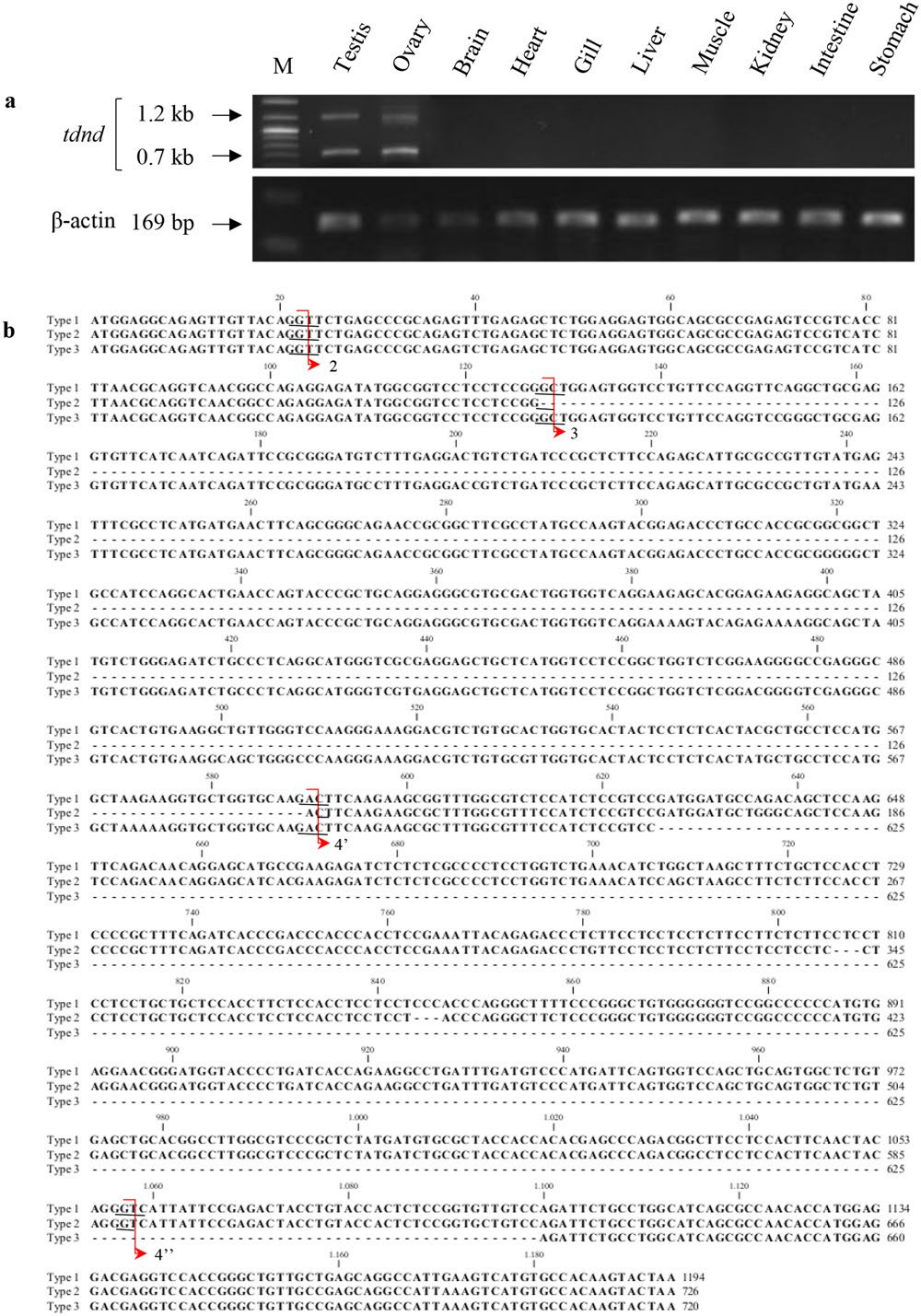
Three types of *tdnd* transcripts. (**a**) RT-PCR of *tdnd* using tissues cDNA, showing different lengths of transcripts. The images were juxtaposed (the full-length gels are included in the Supplementary Information file). M, markers. (**b**) Nucleotide sequence alignment of the three transcripts. Red arrows delimit each exon, with the donor and acceptor sites underlined.

Gel electrophoresis of RT-PCR products showed *tdnd* fragments of different lengths (Fig. 4a). The transcripts were detectable only in the tambaqui’s testes and ovaries and no expression was detected in other tissues, including the heart, brain, gills, liver, muscle, kidneys, intestine and stomach. Further, sequencing of each fragment revealed that *tdnd* has three transcripts, because two smaller fragments were nearly identical in length and could not be distinguished on the electrophoretic pattern. Alignment of the obtained sequences showed that type 1 represents the full sequence of *tdnd*, type 2 does not contain exon 3 (aa 43–196), and type 3 has a missing part of exons 4 and 5 (aa 209–366). The amino acid alignment of the three types of transcripts (Fig. 4b) shows the limit of each exon (red arrows).

**Figure 5:**
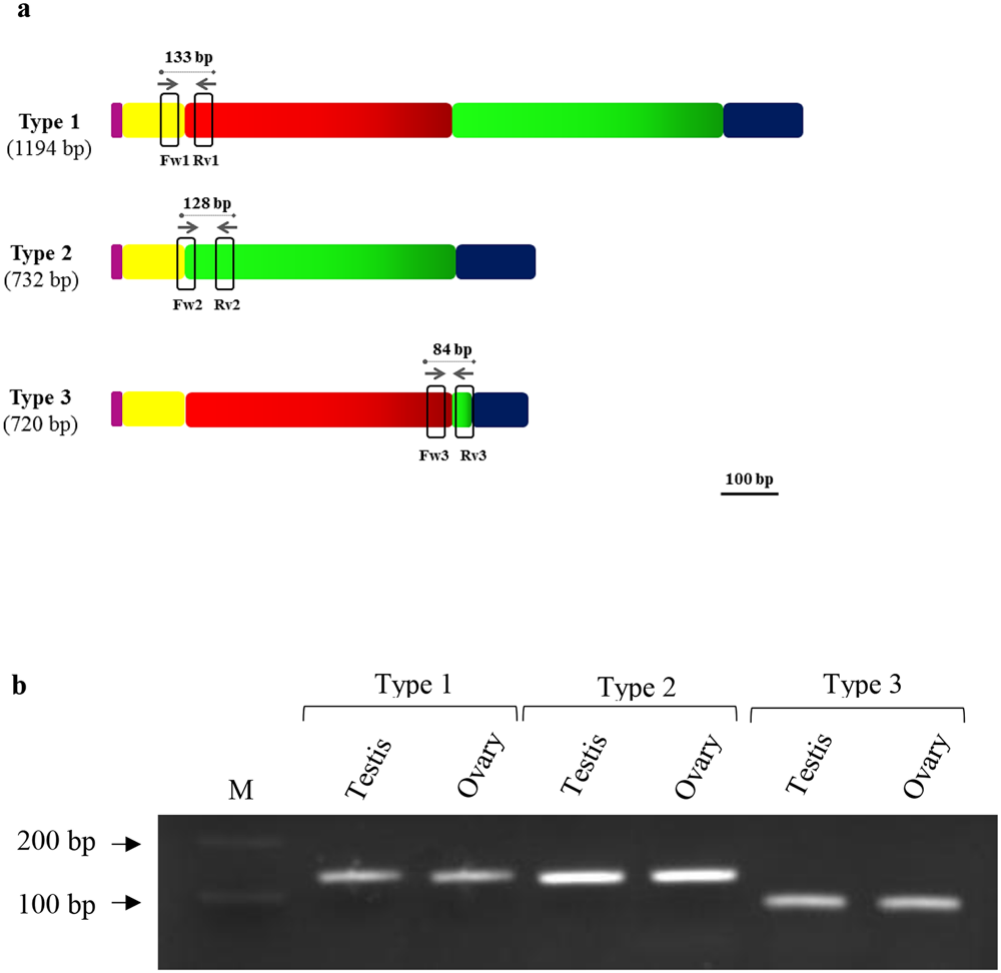
Structure and expression of *tdnd* variants. (**a**) Schematic drawing of the position and length of each specific primer. The different colors represent each exon in the gene. (**b**) Expression of *tdnd* transcripts in the testis and ovary. M, markers.

To determine the tissue distribution profile of each transcript, we designed specific primers (Table 1) to each transcript in order to distinguish them (Fig. 5a). The results showed that all transcripts are detectable in the tambaqui’s testes and ovaries (Fig. 5b).

**Table 1:** Primers designed to detect the *dnd* gene isoforms of *Colossoma macropomum*.

Purpose	Gene	Primer name	Sequence
Cloning	*tdnd*	*tdnd*-deg-Fw-1	5′-GGSTGTGAGGTTTTCATCAGTCAG
		*tdnd*-deg-Rv-1	5′-CACAWGGTTTGGATCACCTGCTCA
5′RACE	*tdnd*	*tdnd*-5RACE-1	5′-GTCGAAGTTCATCATGAGGCGAAACTCATACAA
		*tdnd*-5RACE-2	5′-ACTCATACAACGGCGCAATGCTCTGGAAGA
3′RACE	*tdnd*	*tdnd*-3RACE-1	5′-GATGGTACCCCTGATCACCAGAAGGCCTGA
		*tdnd*-3RACE-2	5′-GAAGGCCTGATTTGATGTCCCATGATTCAGT
RT-PCR	*tdnd*	*tdnd*-total-Fw	5′-ATGGAGGCAGAGTTGTTACAGGTTCTGAG
		*tdnd*-total-Rv	5′-CTTGTGGCACATGACTTTAATGGCCTGCT
RT-PCR and qPCR	*actb*	actin-Fw	5′-CGTGATGGACTCTGGTGATG
		actin-Rv	5′-TCACGGACAATTTCCCTCTC
Alternative Splicing and qPCR	*tdnd*	Fw1	5′-CAGGTCAACAGCCAGAGGAG
		Rv1	5′-GGAAGAGCGGGATCAGACAG
		Fw2	5′-AGAGACCCTGTTCCTCCTCC
		Rv2	5′-ACCATCCCGTTCCTCACATG
		Fw3	5′-CCTCTCACTATGCTGCCTCC
		Rv3	5′-TCTGGACGGAGATGGAAACG
Genomic DNA	*tdnd*	Genomic-Fw	5′-CTTCAAGAAGCGGTTTGGCGTCTCCATCTCC
		Genomic-Rv	5′-TTGGCGCTGATGCCAGGCAGAATCTGGAC

### *tdnd* expression

**Figure 6:**
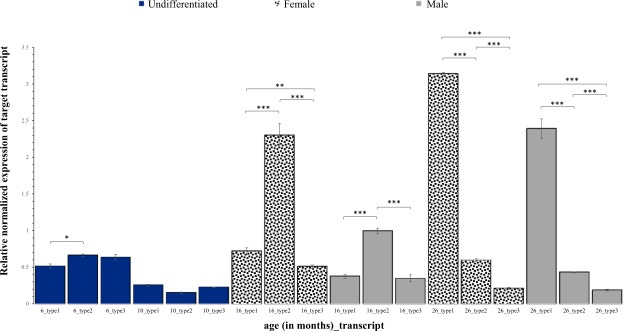
Relative normalized expression of the *dnd* transcripts type 1, 2 and 3 at different ages (in months). All error bars represent standard deviation. Statistical significance: ***p < 0.0005; **p < 0.005; *p < 0.05; n.s.: *p* > 0.05.

Quantitative analysis with testes and ovaries of the tambaqui of different ages showed that the three *dnd* transcripts present distinctive profiles of expression, although the relative quantification varies largely depending on the fish’s gender and age (Fig. 6). The expression profile of the full-length *dnd* transcript (type 1) was distinct from that of the short transcripts (types 2 and 3), including the alternative splicing variant (type 2), and showed the highest expression in the 26-month-old tambaqui.

**Figure 7:**
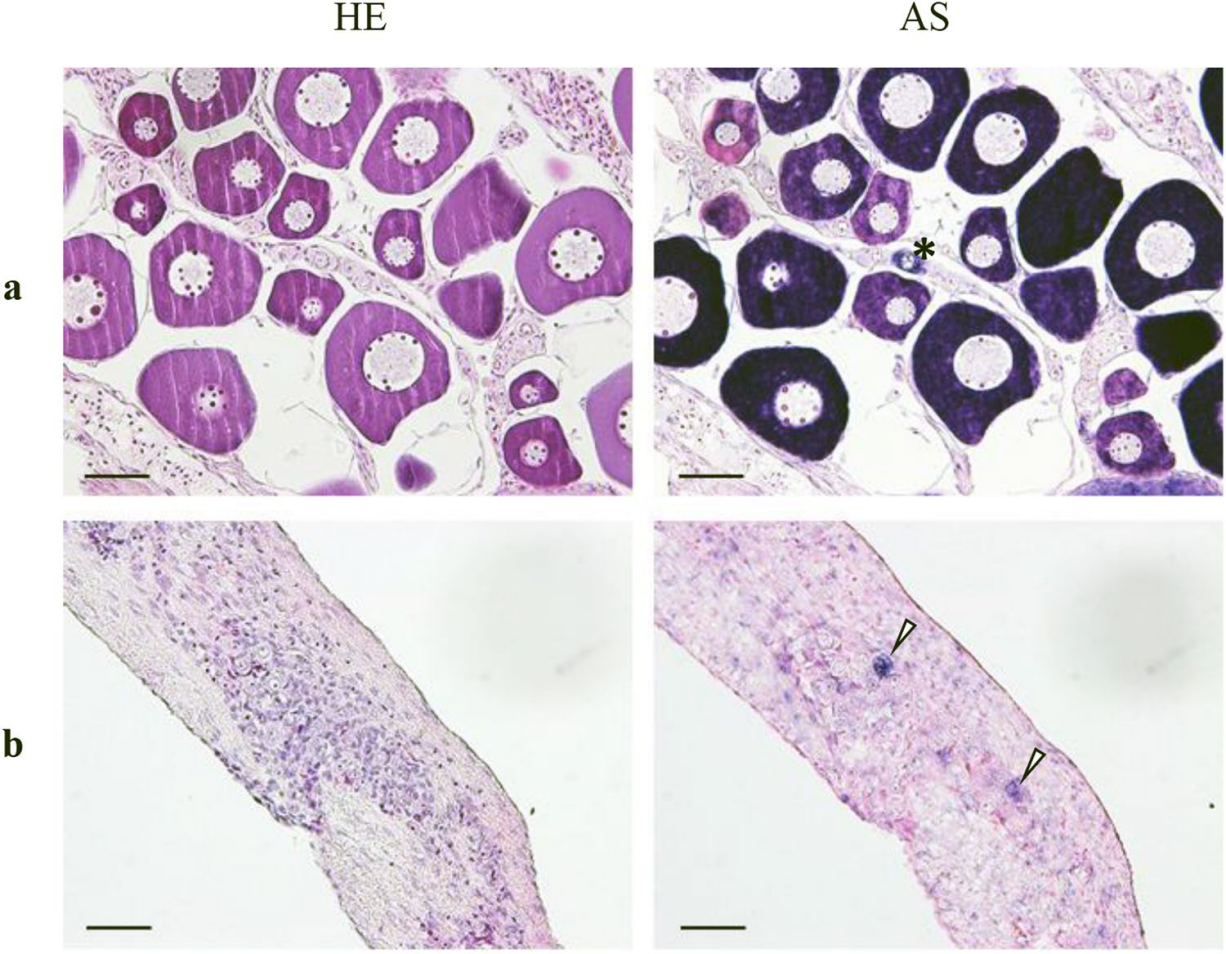
ISH analysis of *tdnd* in gonad sections of the tambaqui. (**a**) Strong expression of *tdnd* was detected in previtellogenic oocytes. Oogonia are shown by asterisks. (**b**) Testicular expression of *tdnd* showed weak signals in some spermatogonia (white arrows). HE, hematoxylin–eosin; AS, antisense probe. Scale bars = 50 µm.

We conducted further investigation of *tdnd* mRNA localization at the cellular level by ISH with type 1 *tdnd* probes on serial sections of tambaqui gonads. In the ovarian section, perinuclear-stage oocytes showed clear, positive signals, while in the testicular section, weak signals were detected in immature spermatogonia (Fig. 7).

## Discussion

In this study, we identified and characterized *dnd* cDNA as a candidate for a germ cell marker in the tambaqui. In addition, we found three transcripts of *tdnd* being expressed in the gonads of both male and female tambaqui. The amino acid sequence deduced from full-length cDNA showed 98% similarity and 53% identity with the zebrafish *dnd*, which has been functionally analyzed by gene knock-down techniques^10^ and *in vitro* intermolecular interaction analyses^31^. Phylogenetic tree analysis of Dnd-related proteins revealed that the clone isolated in this study belongs to the Dnd homolog branch. Furthermore, the deduced amino acid sequence contains two single-stranded RRMs and one DSRM, which are typical characteristics of the Dnd family. These three conserved domains are found in the all known Dnd proteins, including the red piranha (GenBank No. XM_017722544.1), the Mexican tetra (XM_007253600.3), goldfish (JN578697.1), golden-line barbell (XM_016243058.1), common carp (XM_019103334.1), zebrafish (AY225448.1), rainbow trout (DQ974158.1), southern BFT (KP171240.1), Atlantic salmon (JN712911.1), medaka (NM_001164516.1), *Xenopus* (AAX84947.1), and mouse (NP_775559.2). Furthermore, *tdnd* was specifically and strongly expressed in the tambaqui’s oocytes, suggesting that *tdnd* is a germline gene and possibly works as a maternally inherited factor^32^. Thus, at least from structural and expression points of view, results of the sequence homology, phylogenic tree analyzes and domain structure, together with *tdnd*’s specific expression in the germ cell lineage, fulfill the requirements of a *dnd* homolog.

By sequence alignment of genomic DNA and *tdnd*, we revealed that *tdnd* comprises five exons, instead of four, which are typically observed in most of the vertebrates, such as humans (NC_000005.10), mice (NC_000084.6), and zebrafish (NC_007125.7). By comparing the *dnd* exon–intron structure of these species, we found that exon 4 was segmentalized into two exons, producing five exons collectively in the tambaqui. The intron inserted between exons 4 and 5 of *tdnd* (GenBank No. MG879029) has a typical splice donor site (G-G-[cut]-G-U-R-A-G-U) and a splice acceptor site (Y-rich-N-C-A-G-[cut]-G), in addition to the branch sequence Y-U-R-A-C, which is located 20–50 nucleotides upstream of the acceptor site^33,34^. When the exon–intron structure is analyzed in teleosts, we can observe that *dnd* in the cyprinid fish has four exons, whereas *dnd* in other fish species, including the *tdnd*, has five exons. The positions of these additional introns suggest that intron insertion occurs independently in the tambaqui’s *dnd* compared with salmonids and medaka, because the tambaqui has an additional intron in exon 4 and salmonids and medaka have one in exon 3. At this moment, we cannot discuss the evolutionary processes of these events, because the exon–intron structure information of the *dnd* in teleosts is still scarce^35^.

In this study, three types of *tdnd* transcripts were identified. Although alternative splicing occurs with high frequency in pre-mRNA comprising more than one exon^36,37^, we reported for the first time the occurrence of alternative splicing in *dnd*, which generated at least one additional isoform with a different expression profile. Although the recognition of each exon and intron in the tambaqui showed that type 2 is generated from alternative splicing of exon 3, the truncated isoform 3 shows a different pattern of splicing compared with the patterns documented so far^4^.

It is known that alternative splicing is one of the main sources of proteomic diversity in multicellular eukaryotes^9^. Using alternative splicing, mRNAs with different functions can be synthesized from a single gene, although not all alternatively spliced transcripts produce functional proteins^38^. In addition to be a mechanism for proteomic expansion, alternative splicing appears to modulate gene function by adding or removing protein domains, affecting protein activity, or altering transcriptional stability or a resulting protein^39–41^. This process involves the differential use of splice sites to create distinct proteins. Our results showed that isoforms 2 and 3 of *tdnd* are generated by different splice sites. Exon 3 of the type 2 isoform is completely removed by the process termed “exon skipping”^8^ and the loss causes complete exclusion of RRM1 and the loss of 62 RRM2 amino acids (~80% of its composition). In the type 3 transcript, 7 amino acids (9%) of RRM2 and 51 amino acids (64%) of the DSRM are excluded from mature mRNA during RNA processing. Although studies have reported that some RNA-binding proteins may lose the RRM but retain function through other well-conserved and structurally important residues^42^, RRM loss in transcripts 2 and 3 may inactivate the protein, since the domains play a crucial role in *dnd* function.

Alternative splicing in *tdnd* may be related to down-regulation of gene function^43–49^, such as dominant negative inhibition. This is because the absence of the above-mentioned motifs, which are important for the protein function, may impair recognition of the molecule, causing the loss of *dnd*’s protective function against miRNA, which acts as a post-transcriptional silencer. This protection function is due to *dnd*’s interaction with the mRNA molecule via neutralization of the function of several miRNAs in the PGCs, preventing them from associating with their target sites^11^. Furthermore, in the sexually mature tambaqui, we observed that the relative expression level of isoform 1 is clearly distinct than the splicing variants 2 and 3, showing a possible inhibition of transcription caused by the other isoforms in 16-month-old animals. Therefore, we proposed that the production mechanism of alternative forms of *dnd* transcripts in the tambaqui is regulated differently from that of the full-length transcript (type 1).

In any case, specific expression of all three variants in the gonads may suggest their importance in germline development. Because we could not distinguish each variant by using the RNA probe in the ISH study, further, precise analysis is needed to determine the specific expression of each transcript in the germ cell lineage.

Future studies aiming to preserve the genetic variability of tambaqui should consider the expression of the molecular marker *tdnd*, since the greater expression of the functional form of the gene found in the gonads represents a greater amount of germ cells. If the splice variants do indeed regulate the functional isoform of the gene in tambaqui, donors with a greater expression of the spliced transcripts should be avoided in a germ cell transplantation study.

## Methods

### Ethical statement

All experimental protocols employed in the present study were approved by the Administrative Panel on Laboratory Animal Care and Use at Tokyo University of Marine Science and Technology (Japan), and by the Research Ethics Committee of the Federal University of Rio Grande do Sul (Brazil). All methods were carried out in accordance with the Guide for the Care and Use of Laboratory Animals from Tokyo University of Marine Science and Technology.

The samples were acquired from a commercial hatchery and were not subjected to any experimental manipulation.

### Fish materials and RNA isolation

The *Colossoma macropomum* subjects used in this study were collected at a fisheries farm located in the Rondônia state, in the northwestern region of Brazil (Supplementary Table S2). The samples from 6-, 10-, 16- and 26-month-old tambaqui were harvested immediately after euthanasia by an overdose of MS-222 and analyzed individually. Fragments of the ovary, testis, heart, brain, gills, liver, muscle, kidney, intestine and stomach were immersed in RNAlater® Solution (Thermo Fisher Scientific) and stored at −80 °C until further use. Total RNA was extracted from the tissues using a QuickPrep Total RNA Extraction Kit (Amersham Pharmacia Biotech, NJ, USA). We examined the quality of the isolated RNA by a spectrophotometer and denaturing gel electrophoresis.

### RT-PCR

We used 3 µg of total RNA for complementary deoxyribonucleic acid (cDNA) synthesis. First-strand cDNA was synthesized by means of Ready-To-Go You-Prime First-Strand Beads (Amersham Pharmacia Biotech) with the adapter-oligo dT primer (5′-CTGATCTAGAGGTACCGGATCC-oligo dT-3′). Reverse transcription–polymerase chain reaction (RT-PCR) was performed with degenerate primers (*dnd*-Fw1 and *dnd*-Rv1), which were designed using highly conserved regions of *dnd* homologs from various fish species, and the ß-actin was used as an internal control for tissue-specific gene expression. PCR was performed using 1 μL of cDNA products, 0.8 μL of deoxyribonucleotide triphosphate (dNTP) Mixture (2.5 mM each), 1 μL of each primer (10 μM), 1 μL of 10x ExTaq buffer (Takara Biomedicals), and 0.25U of ExTaq enzyme (Takara Biomedicals), in a total volume of 10 μL. Amplification was performed with an initial denaturation step of 5 min at 94 °C, followed by 35 cycles as follows: (i) denaturation for 30 s at 94 °C, (ii) annealing for 30 s at 63.9 °C, and (iii) extension for 1 min at 72 °C. The final extension was performed for 3 min at 72 °C. PCR products were electrophoresed on a 0.7% agarose gel, and the DNA fragment that showed the predicted molecular weight was isolated using a Gelpure DNA Purification Kit (GeneMate). Amplified cDNA fragments were cloned into the pGEM T-Easy Vector (Promega, Madison, WI, USA) and sequenced as described before^50^. Table 1 shows all the primers used.

### RACE

To isolate a full-length cDNA, using gene-specific primers for 5′ rapid amplification of cDNA ends (RACE; *tdnd*-5′RACE-1 and *tdnd*-5′RACE-2) and 3′RACE (*tdnd*-3′RACE-1 and *tdnd*-3′RACE-2), we performed 5′RACE and 3′RACE using a GeneRacer Kit (Life Technologies Corporation, Carlsbad, CA, USA) according to the manufacturer’s instructions. Then, we cloned amplified RACE products into the pGEM T-Easy Vector (Promega) and sequenced. We used touchdown and nested PCR to reduce background amplification and increase the specificity and sensibility of RACE products.

### Phylogenetic analysis

To ensure that the genetic sequence obtained belongs to the *dnd* branch, we designed a phylogenetic tree comparing the deduced amino acid (aa) sequences of the *dnd* cDNAs from various species. The alignment of each gene was generated by MAFFT version 7 with the L-INS-i method^51^ and the aa gapfree sites from each alignment were then used for maximum likelihood phylogenetic analyses carried out using PhyML v3.0 server^52^. The protein evolutionary model of *vasa* was selected as JTT + G + I + F by Smart Model Selection (SMS) with Akaike’s information criterion (AIC)^53^. The reliability of the tree was tested by bootstrap resampling with 1000 replicates. The a1cf, related protein, was included in the analysis and assigned as the outgroup.

### Histology and ISH

Fragments of tambaqui testes and ovaries were fixed with Bouin’s solution, cut into 4-µm-thick sections using standard paraffin-embedding methods, and stained with hematoxylin and eosin. We analyzed localization of *tdnd* mRNA by ISH on the sections, as described in a previous study^28^. A 1194 base pair (bp) cDNA fragment of *tdnd* was used as a template to synthesize an antisense RNA probe.

### Genomic DNA analysis

To study the alternative splicing of *tdnd* type 3, we extracted genomic DNA using a Gentra Puregene Tissue Kit (QIAGEN, Hilden, Germany) according to the manufacturer’s instructions. Next, we amplified genomic DNA encoding *tdnd* by PCR using 1 μL of genomic DNA products, 0.8 μL of dNTP Mixture (2.5 mM each), 1 μL of each primer (10 μM), 1 μL of 10x ExTaq buffer (Takara Biomedicals), and 0.25 units of ExTaq enzyme (Takara Biomedicals), in a total volume of 10 μL. Then, amplification was performed with an initial denaturation step of 5 min at 94 °C, followed by 35 cycles as follows: (i) denaturation for 30 s at 94 °C, (ii) annealing for 30 s at 66 °C, and (iii) extension for 1 min at 72 °C. The final extension was performed for 3 min at 72 °C. PCR products were electrophoresed on a 0.7% agarose gel, and the DNA fragment that showed the predicted molecular weight was isolated using a Gelpure DNA Purification Kit (GeneMate). Amplified genomic DNA fragments were cloned into the pGEM T-Easy Vector (Promega) and sequenced. Table 1 shows the primers used.

### Quantitative PCR (qPCR)

To compare the expression profiles of each isoform at different ages of the tambaqui, we amplified the cDNA in triplets (three technical replicates) per gene and biological replicate (sample) using intercalating dye SsoAdvanced Universal SYBR Green Supermix Kit (Bio-Rad Laboratories, Inc., Hercules, CA, USA), following the manufacturer’s instructions. Quantifications of *tdnd* types 1, 2 and 3 were performed by the comparative Ct (ΔΔCt) method and normalized using the *actb* as reference gene. Each reaction was performed using 1 μL of cDNA products, 0.2 μL of each primer (10 μM), 5 μL of SsoAdvanced Universal SYBR Green Supermix (2x), and 3.6 μL of nuclease-free water, in a total volume of 10 μL. Termocycler program consisted of an initial hot start cycle at 95 °C for 30 sec, followed by 40 cycles at 95 °C for 15 sec and 60 °C for 30 sec. To confrm product specifcity, melting curve analysis was performed afer each amplifcation, consisting of a dissociation step cycle at 65 °C for 5 sec then 0.5 °C for 10 sec until 95 °C. The existence of one peak in melting curve analysis was used to confirm gene-specific amplification and to rule out non-specific amplification and primer-dimer generation. Relative standard curves for the transcripts were generated with a serial dilution of cDNA.

### Statistical analyses

Statistical analyses were carried out using StatPlus Pro 6.4. Datasets were compared using one-way ANOVA followed by Tukey-Kramer post-hoc analyses. Tissue expression results were expressed as mean normalized ratios (±SE) corresponding to the ratio between the copy numbers of the target transcript (*dnd* type 1, type 2 and type 3) and the copy numbers of the reference gene, *actb*. Statistics are represented in the figures by asterisks and results were considered significant if p < 0.05 (*p < 0.05, **p < 0.05, ***p < 0.005).

## Supplementary information

Supplementary information
