# Supplementary material for: The germ cell marker dead end reveals alternatively spliced transcripts with dissimilar expression
Source: Sci Rep. 2019 Feb 20;9:2407. doi: 10.1038/s41598-019-39101-9 (PMC6382762; doi:10.1038/s41598-019-39101-9)
Supplement: Supplementary file 1 — Supplementary information [file 41598_2019_39101_MOESM1_ESM.docx]

**The germ cell marker *dead end* reveals alternatively spliced transcripts with dissimilar expression**

Ana Carina Nogueira Vasconcelos^*^, Danilo Pedro Streit Jr, Anna Octavera, Misako Miwa, Naoki Kabeya, Goro Yoshizaki

Supplementary information

Table S1. The identities of tambaqui Dnd to other Dnd proteins.

| **Species** | **Identity** | **Accession** | **Species** | **Identity** | **Accession** |
| --- | --- | --- | --- | --- | --- |
| *Pygocentrus nattereri* | 84% | [XP_017578033.1](https://www.ncbi.nlm.nih.gov/protein/XP_017578033.1?report=genbank&log$=prottop&blast_rank=3&RID=WWB1SUFT014) | *Salmo salar* | 48% | [NP_001266060.1](https://www.ncbi.nlm.nih.gov/protein/NP_001266060.1?report=genbank&log$=prottop&blast_rank=28&RID=WWB1SUFT014) |
| *Astyanax mexicanus* | 68% | [XP_007253662.2](https://www.ncbi.nlm.nih.gov/protein/XP_007253662.2?report=genbank&log$=prottop&blast_rank=4&RID=WWB1SUFT014) | *Xenopus tropicalis* | 47% | NP_001037899.1 |
| *Ictalurus punctatus* | 59% | XP_017340221.1 | *Thunnus orientalis* | 47% | [AHB61249.1](https://www.ncbi.nlm.nih.gov/protein/AHB61249.1?report=genbank&log$=prottop&blast_rank=55&RID=WWB1SUFT014) |
| *Carassius auratus* | 57% | [XP_026080930.1](https://www.ncbi.nlm.nih.gov/protein/XP_026080930.1?report=genbank&log$=prottop&blast_rank=6&RID=WWB1SUFT014) | *Oryzias latipes* | 46% | [NP_001157988.1](https://www.ncbi.nlm.nih.gov/protein/NP_001157988.1?report=genbank&log$=prottop&blast_rank=59&RID=WWB1SUFT014) |
| *Sinocyclocheilus grahami* | 56% | [XP_016098544.1](https://www.ncbi.nlm.nih.gov/protein/XP_016098544.1?report=genbank&log$=prottop&blast_rank=8&RID=WWB1SUFT014) | *Mus musculus* | 45% | NP_775559.2 |
| *Cyprinus carpio* | 56% | [XP_018958879.1](https://www.ncbi.nlm.nih.gov/protein/XP_018958879.1?report=genbank&log$=prottop&blast_rank=10&RID=WWB1SUFT014) | *Homo sapiens* | 45% | NP_919225.1 |
| *Danio rerio* | 53% | [NP_997960.1](https://www.ncbi.nlm.nih.gov/protein/NP_997960.1?report=genbank&log$=prottop&blast_rank=20&RID=WWB1SUFT014) | *Crocodylus porosus* | 42% | NP_019390319.1 |
| *Oncorhynchus mykiss* | 48% | [CDQ77433.1](https://www.ncbi.nlm.nih.gov/protein/CDQ77433.1?report=genbank&log$=prottop&blast_rank=23&RID=WWB1SUFT014) | *Parus major* | 42% | NP_015497053.1 |

Table S2. Overview of the *Colossoma macropomum* used, including anatomical, morphological and physiological characteristics of the animals.

| **Sample** | | **Age (months)** | **Body weight (Kg)** | **Body length (cm)** | | **Gender** | |
| --- | --- | --- | --- | --- | --- | --- | --- |
| 1 | 6 | | 0.057 | | 15.5 | | undifferentiated |
| 2 | 6 | | 0.047 | | 15.0 | | undifferentiated |
| 3 | 6 | | 0.046 | | 14.5 | | undifferentiated |
| 4 | 6 | | 0.042 | | 14.0 | | undifferentiated |
| 5 | 10 | | 0.540 | | 25.0 | | undifferentiated |
| 6 | 10 | | 0.685 | | 28.0 | | undifferentiated |
| 7 | 10 | | 0.630 | | 26.5 | | undifferentiated |
| 8 | 10 | | 0.650 | | 27.0 | | undifferentiated |
| 9 | 16 | | 0.843 | | 35.5 | | Female |
| 10 | 16 | | 0.942 | | 36.5 | | Female |
| 11 | 16 | | 1.092 | | 38.5 | | Female |
| 12 | 16 | | 0.789 | | 35.0 | | Male |
| 13 | 16 | | 0.802 | | 34.5 | | Male |
| 14 | 16 | | 0.750 | | 33.0 | | Male |
| 15 | 26 | | 2.600 | | 43.0 | | Female |
| 16 | 26 | | 3.600 | | 48.0 | | Female |
| 17 | 26 | | 3.750 | | 51.0 | | Female |
| 18 | 26 | | 2.250 | | 44.0 | | Male |
| 19 | 26 | | 2.200 | | 43.0 | | Male |
| 20 | 26 | | 2.100 | | 43.5 | | Male |
| 21 | 26 | | 2.900 | | 45.0 | | Male |
